# Indoor Location Data for Tracking Human Behaviours: A Scoping Review

**DOI:** 10.3390/s22031220

**Published:** 2022-02-05

**Authors:** Leia C. Shum, Reza Faieghi, Terry Borsook, Tamim Faruk, Souraiya Kassam, Hoda Nabavi, Sofija Spasojevic, James Tung, Shehroz S. Khan, Andrea Iaboni

**Affiliations:** 1 KITE—Toronto Rehabilitation Institute, University Health Network, Toronto, ON M5G 2A2, Canada; leia.shum@uhnresearch.ca (L.C.S.); reza.faieghi@ryerson.ca (R.F.); terry.borsook@uhnresearch.ca (T.B.); tamim.faruk@mail.utoronto.ca (T.F.); souraiya.kassam@mail.utoronto.ca (S.K.); hoda.nabavi@uhnresearch.ca (H.N.); sofija.spasojevic@uhn.ca (S.S.); shehroz.khan@uhn.ca (S.S.K.); 2 Department of Aerospace Engineering, Ryerson University, Toronto, ON M5B 2K3, Canada; 3 Institute of Biomedical Engineering, University of Toronto, Toronto, ON M5S 3G9, Canada; 4 Department of Mechanical and Mechatronics Engineering, University of Waterloo, Waterloo, ON N2L 3G1, Canada; james.tung@uwaterloo.ca; 5 Department of Psychiatry, University of Toronto, Toronto, ON M5T 1R8, Canada

**Keywords:** computational intelligence, data analytics, digital phenotyping, health monitoring technologies, human behaviour, real-time location systems, sensor-based assessments

## Abstract

Real-time location systems (RTLS) record locations of individuals over time and are valuable sources of spatiotemporal data that can be used to understand patterns of human behaviour. Location data are used in a wide breadth of applications, from locating individuals to contact tracing or monitoring health markers. To support the use of RTLS in many applications, the varied ways location data can describe patterns of human behaviour should be examined. The objective of this review is to investigate behaviours described using indoor location data, and particularly the types of features extracted from RTLS data to describe behaviours. Four major applications were identified: health status monitoring, consumer behaviours, developmental behaviour, and workplace safety/efficiency. RTLS data features used to analyse behaviours were categorized into four groups: dwell time, activity level, trajectory, and proximity. Passive sensors that provide non-uniform data streams and features with lower complexity were common. Few studies analysed social behaviours between more than one individual at once. Less than half the health status monitoring studies examined clinical validity against gold-standard measures. Overall, spatiotemporal data from RTLS technologies are useful to identify behaviour patterns, provided there is sufficient richness in location data, the behaviour of interest is well-characterized, and a detailed feature analysis is undertaken.

## 1. Introduction

Real-time location tracking systems (RTLS), or indoor positioning or location systems, are primarily used for tracking individuals and equipment in indoor environments in real or near-real time [1]. Over time, these systems accumulate information about the movement of individuals and are thus a valuable source of longitudinal spatiotemporal data, which can be used to help understand patterns of human movement and behaviour. RTLS systems have privacy-preserving advantages over other technologies [2] and are relatively low-cost and easy-to-use compared to many wearable sensor technologies [3]. An advantage of indoor location data over outdoor monitoring (such as using GPS) is the ability to characterize movement through a well-defined target environment and extrapolate insights about the purpose or pattern of movement in that environment.

RTLS can be realized using various sensor technologies including Bluetooth, ultra-wideband (UWB), and passive infrared (IR) sensors. Considering that accuracy and sampling rate for each technology is different, the degree of information that can be inferred from each RTLS technology varies. In general, RTLS provides two or three dimensions of spatial movement data over a recorded period of time and can be provided either uniformly at a set sampling frequency or through passively triggered events. Location data from RTLS systems are sometimes collected in conjunction with other sensor data such as biometric (heart rate, oxygen, galvanic skin response) or movement (inertial measurement units (IMUs), body joint and angle tracking through video or depth) data. By combining location information with other sensor data, it is possible to recognize behaviours or events with higher degree of certainty (i.e., using acceleration plus immobility to identify the occurrence of a fall). Different types of features extracted from RTLS data can be used to infer patterns of human behaviour for different applications. For example, walking distance or speed can be used as an indicator of physical activity level, or proximity to others as a measure of social interaction. Many existing RTLS monitoring systems used in healthcare settings already provide location data from which long-term records of patterns of behaviour can be extracted as objective indicators of health status. The number of studies that have explored the application of RTLS for health status monitoring has been growing over the past few years. These studies have focused on the use of RTLS data for assessing clinical symptoms or measures, such as gait performance [4], mood, apathy, depression [5], cognitive decline [6], and the onset of dementia [7]. RTLS technologies are widely used for clinical applications in healthcare settings, including in hospitals and residential care homes [4,5,6,8,9]. Current real-time applications for RTLS systems include elopement prevention and for staff to locate patients. These existing sources of RTLS data represent an important opportunity to develop low-cost and low-effort health status monitoring technologies for older adults.

Presently, there are a small number of review papers regarding the applications of RTLS in healthcare [1,10]. However, these papers focus on the feasibility and user-acceptance of the technology rather than examining approaches to analyzing RTLS data to describe human movement and behaviour. Moreover, existing technical reviews of RTLS technology focus on physical implementation challenges or describe configurations and accuracy of available hardware used [11,12]. Understanding features of spatiotemporal tracking data and analytic approaches are valuable in describing and assessing patterns of human behaviours. These descriptions and assessments can help to expand the analytic methods that can be applied to RTLS and help inform which approaches will best suit each setting and use case. Given the wide variety of applications of RTLS, there is a need to consolidate the existing knowledge and outline the potential of RTLS for health status monitoring among different application domains.

RTLS has growing applications beyond healthcare, in fields where understanding human behaviour is important, for example, education [13,14], retail [15], transportation [16], and construction [17,18]. As these domains provide a new set of examples for applications of RTLS in behavioural assessments, understanding the state of the art in these domains can potentially lead to the generation of new ideas in the development of RTLS-based health status monitoring systems. With this in mind, this scoping review will cover literature from a wide range of domains including healthcare and related fields. We chose a scoping review approach, as guided by the PRISMA-ScR extension [19], to address our broad research question by providing an overview of literature in this area and allowing the inclusion of studies with a variety of different study designs and methods. In this paper, we present the results of our scoping review with the following research question: How are human behaviours and their patterns described using real-time indoor location data? In answering the above question, this paper will describe: (1) the types of behaviours, symptoms, and measures that have been described using RTLS technologies; (2) approaches to analysis of RTLS data and categories of features that can be extracted from RTLS measurements; and (3) how RTLS data and its analysis varies by the type of RTLS technology used.

## 2. Methods

### 2.1. Search Strategy

The Scopus database was used to perform a comprehensive search to find papers that analysed human behaviours using RTLS technology. The search terms consisted of three different concepts of location AND indoor AND behaviour as described further in Table 1. The search was conducted for all research accepted/published in Scopus before 31 March 2021.

### 2.2. Study Selection

Results from the Scopus search were imported to Covidence, a literature review tool [20], for initial screening and deduplication of database results. Two reviewers (RF, TB) independently screened article titles and abstracts and a third reviewer (AI) resolved any disagreements. This process was repeated for full-text screening. In keeping with a scoping review methodology, no formal quality assessment of the studies was undertaken.

Papers were included if they were found to contain the following criteria: (1) written in or translated into English, (2) containing experimental datasets from human participants, and (3) using location data from RTLS technologies to describe a facet of human behaviour.

Papers that focused on RTLS implementation or were proof of concept without presenting any data were excluded. Additionally, papers were excluded if they were reviews, were not peer-reviewed, only used location of joints relative to the human body, performed only technological validation of location tracking or analyzed the accuracy of the localization technology, included only outdoor or vehicular tracking, or examined the flow of movement in a location without linking this movement to a behaviour, symptom, or measure from a single individual.

### 2.3. Data Extraction

We extracted study population information (e.g., sample size, clinical population characteristics, inclusion criteria), technical device details (e.g., sensor type, accuracy, vendor), study behaviours/symptoms and measures, RTLS features, analysis methods, and findings. Six reviewers (RF, TB, SK, SM, HN, SS, AI) extracted data from the selected papers, with each paper reviewed twice, and three key authors (RF, TF, LS) resolving any conflicts that occurred between the two rounds of review for each paper. For the purposes of this study, a feature was defined as a distinct individual property or characteristic [21] measurable using a set of recorded locations from RTLS data.

An iterative approach to reporting results was taken: two key authors (TF, LS) consolidated results and reported to the study team for insights, revisions, and refinement. The results from data extraction were then discussed and a framework was developed for key foci: general study population and description, focal areas of study outcome measures, common features extracted from RTLS data, and technical aspects surrounding the different RTLS technology used with the selected papers. Through discussion and consensus with a third author (AI), the selected papers were categorised based on their study field.

## 3. Results

### 3.1. Search Results

**Figure 1:**
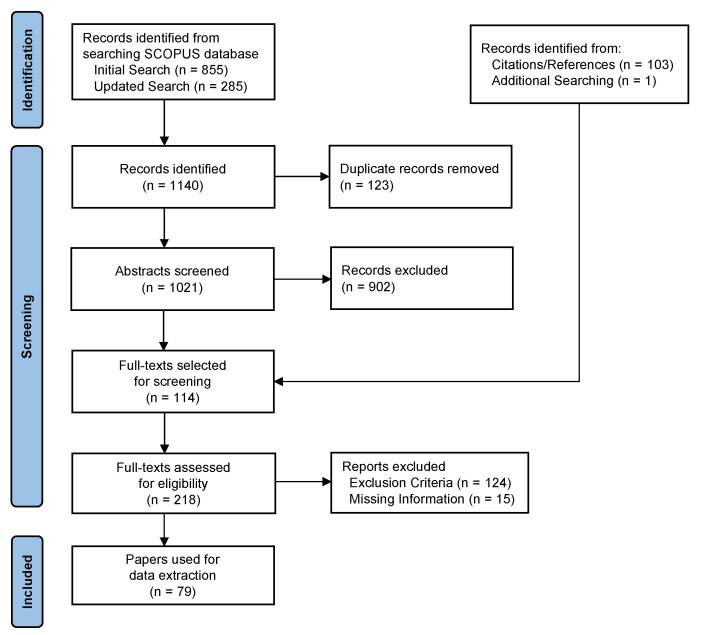
Flow of sources of literature through the paper screening process.

The database search yielded a total of 1140 papers. After removing 123 duplicates detected with the Covidence tool, paper and title abstracts were screened using the study selection criteria presented in Section 2.2. An initial 114 papers were included in the full text review, and an additional 103 papers were later added from citation and reference searching of these included papers. A total of 218 full-texts were thus reviewed to confirm if they met the inclusion or exclusion criteria used for abstract screening. A total of 79 papers met these criteria and were included in the data extraction and analysis. Figure 1 describes the flow of papers collected for screening.

### 3.2. Application Sectors and Study Populations

The included papers were sorted into four categories of study application: health status monitoring and assessment (60.8%), consumer behaviours for marketing and shopping applications (26.6%), safety and operational efficiency (10.1%), and developmental behaviours in children (2.5%). In order to explore the types and patterns of behaviours that have been described using RTLS technologies, general information such as the studies’ population sizes and demographics were consolidated. Table 2 presents basic study information for the final 79 papers.

The study population varied greatly based on application. The health status monitoring category included sample sizes from 1 to 154 participants, with a median sample size of 15. The consumer behaviour category produced the largest sample sizes, ranging from 180 to 24,452 participant samples. Older adults were the focus of 37 studies, making up 77% of the health monitoring studies. In particular, 21 of the health status monitoring studies (43.8%) examined individuals with cognitive impairment or dementia.

Participants’ private homes were the most common location for data collection at 26.6% of all the papers analysed. Of the largest application type (health status monitoring, *n* = 48 studies), 21 studies (43.8%) installed devices in private homes, followed by 18 studies (37.5%) in nursing homes, assisted living and retirement communities, and 5 studies (10.4%) in hospital inpatient units.

Papers that analysed behaviours in hospital environments made up 10.4% (five studies) of health status monitoring studies and 37.5% (three studies) of workplace safety and efficiency studies. Consumer behaviours were analysed in supermarket (14 studies), shopping mall (2 studies), and museum (5 studies) environments. Both developmental behaviour studies collected data in school playground and classroom settings.

Half of the studies included older adult participants, all of which were in the health status monitoring sector. Of the 48 papers on health status monitoring, 21 (43.8%) included individuals with cognitive impairment.

### 3.3. Types of Symptoms and Measures

Categorizing the study goals and outcome measures provides important descriptive information about the types of behaviour patterns commonly observed when using RTLS data. Across all studies, 28 (35.4%) were found to have at least one “classification” objective in which the study populations’ behaviours were classified or grouped into defined categories of behaviour types. Within the Health Status Monitoring group, 14 studies (29.2%) had a component of classification, such as the classification of cognitive impairment as mild to severe. Classification approaches were found in 11 studies of Consumer Behaviours (e.g., classifying shoppers as impulse or scheduled shoppers) and 3 studies of Operation Efficiency studies (e.g., classifying behaviours as risky/unsafe or safe). Other objectives included (1) a characterization of a population based on their location or movement, e.g., [24,93]; (2) correlation of an environmental or external context-based variable [53,83,86]; (3) support of an observational measure or standard [23]; and (4) development a system that predicts future events or behaviours [63,69]. A notable sub-group (11 studies) of classification-type objectives within the health status monitoring studies was the classification of groups based on risk of specific health deficits, such as cognitive impairment or dementia.

**Figure 2:**
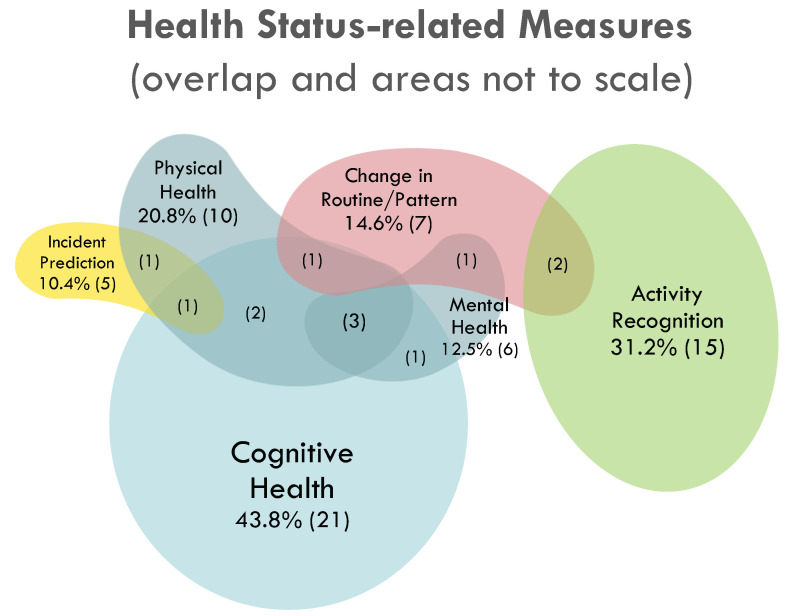
Behavioural outcomes in studies using RTLS for health status monitoring. The percent (number) of studies in each outcome category are provided, and the number of overlapping studies are provided in brackets in the areas of overlap.

Measures of behaviour or outcomes extracted from health status monitoring studies were categorized and presented in Figure 2. Within this group, 13 studies (27.1%) did not pursue outcomes beyond activity recognition. These papers focused on using RTLS data to categorize the specific activity the study subject was performing, with the long-term goal of being able to track patterns in activities for health status monitoring. More studies focused on measuring cognitive health (*n* = 22, 43.8%) over physical (*n* = 11, 20.8%) or mental health (*n* = 7, 12.5%). Of the nine studies that examined a combination of two or more of these health statuses, all contained a cognitive health status component. The detection of changes in behaviour patterns based on participants’ previous RTLS data-generated routines was examined in seven studies (14.6%). Of these seven studies, two [28,49] also attempted to assess physical, cognitive, or mental health status, as shown by the minimal two-paper overlap of “Change in Routine/Pattern” and other health assessment categories in Figure 2.

Within the health status monitoring studies, 21 (43.8%) referenced correlating or validating RTLS-based measures with standardized clinical assessments, such as [23] using the mini-mental state exam (MMSE) as a measure of cognitive impairment. Outside of the health status monitoring category, one Safety and Operational Efficiency study used a standardized assessment (Positive and Negative Affect Schedule (PANAS)) to correlate RTLS data with staff wellness [85] and one Consumer Behaviour study used the RTLS data as a ground truth for generating simulated walking pattern models [81].

All of the consumer behaviour studies (*n* = 16) either used location tracking with the goal of improved visitor experience (*n* = 6) or improved sales (*n* = 15), 12 of which used sales as their primary outcome while varying either store layout or comparing different groups of customers. The remaining four papers studying behaviours in shoppers used the location data of shoppers to develop “shopper type” profiles [68], predict customers’ path [69], and infer information of shopping styles based on population demographics [64,65]. The five studies collecting RTLS data from museum visitors focused on exhibit engagement and/or overcrowding.

The two developmental behaviour studies in children focused on social behaviour outcomes, specifically measuring levels of social interaction between children, and in particular, differences based on gender, and sociability. As these two studies analysed mostly interactions between multiple participants, the results focused on trends of behaviours for predefined groups of participants—in contrast to using models to define the groups or social levels themselves. Of the workplace safety studies, two measured the prediction of hazards based on pre-defined risk factors, three measured work intensity and stress level based on location and time of day, two measured congestion and collisions, and one used behavioural patterns from RTLS data to identify individuals as employees.

### 3.4. Location-Based Features and Analyses

Choosing key features to extract from the location data is an important process that allows the data to be used in a contextually meaningful manner. This review examined existing approaches used in studies and how different kinds of features are used to describe different types of human behaviours. The features of RTLS data used in the analysis of human behaviours in the included papers were categorized into four main groups in a framework based on the complexity and number of components of space and time used:

- Proximities—features using spatial placement of multiple study subjects and how long they were in a measured proximity to each other (two or more locations at a time);
- Trajectory—features indicating direction of movement or generate vectors in 2D space based on a combination of recorded locations (two dimensions of space in time);
- Activity Level—features measuring amount and/or intensity of movement during a period of time (e.g., time spent walking, count of total spaces in which time was spent, number of motion events) (multiple measures of time);
- Dwell Time—features measuring a period of time in specified locations or comparison of duration in pre-defined activities (one measure of time).

**Figure 3:**
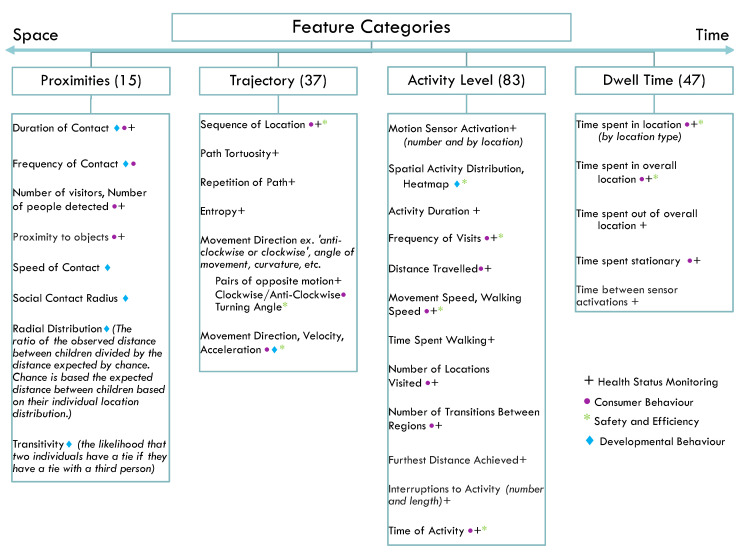
Breakdown of the feature categories and a list of the features observed within each category. The symbols described in the legend represent a binary indicator of whether one or more papers from the application sector denoted used the listed feature. Detailed results for each feature category with study references can be found in supplementary files.

Of the 79 papers included, more than half (48, 60.8%) of the studies included activity level features, closely followed by 38 (48.1%) papers with dwell time features, 35 (44.3%) with trajectory features, and finally 8 (10.1%) papers with features of proximity. Many studies (37, 46.8%) used multiple feature types. Figure 3 presents the feature categories and subgroups of features found within the papers. Of the five studies that include proximity features, there were three studies monitoring health status, one consumer behaviour study, and both papers studying developmental behaviours had proximity-type features.

Two studies [34,35] did not directly define or extract features from the location data, but instead applied a deep convolutional neural network (DCNN) that included feature extraction innately within layers of the neural network. Other papers from the same group also used unique features, such as measuring entropy of movement patterns [38], and extracting abstract features from a binary “activity image” corresponding to location maps and whether location was tracked in each point on the map [39,50]. Another study [43] used RTLS data to provide location context for hand movement accelerometer data, which was then used to develop Random Forest models for activity recognition.

The number of defined features ranged from 1 to 7 kinds of basic features (i.e., features described in Figure 3), in which two features was the most common (36.8%) and median number of features used. Three papers [34,35,43] were excluded in this observation as they did not include features that were defined as spatiotemporal data describing human behaviour. A total of 41 of 79 (51.9%) studies further formulated or abstracted ratios and features derived from their basic spatiotemporal features to analyse outcomes and build models.

Many different analytic approaches were applied to use the RTLS-based features to describe human behaviour. It was observed that 41 (51.9%) studies used at least one machine learning methodology; 22 (27.8%) studies used statistical comparisons; and 6 (7.6%) provided descriptive results using visualizations of location data. The most common machine learning models used were support vector machine (SVM), random forest, and various types of linear and logistic regression modelling. The majority of the machine learning studies (22 out of 41) had a classification goal. Eight (8) studies used machine learning to recognize a defined list of daily activities from location data.

While neither developmental behaviour study used classical machine learning, advanced network science techniques were applied to analyse individual and group behaviours and their interactions. By defining participants as nodes and their proximity features as linked, some techniques used include visualizing social networks and transitivity, subgroup discovery (data mining technique), centrality, and page rank.

### 3.5. Types of Sensors and Technological Systems

The type of sensors and systems used in the selected studies is a key determinant to which features and behaviours can be observed from location-based data. The type of sensors used can affect the data quality, granularity, and possible information extracted. IR sensors were the most commonly used (28; 35.4%), and all IR studies were in the health status monitoring category. Bluetooth (14; 17.7%) and RFID sensors (14; 17.7%) were the next most common, followed by ultra-wide band (UWB) (12; 15.2%). Of the studies that used RFID sensors, 13 were studying consumer behaviours and were often attached to shopping carts and baskets as opposed to directly onto participants. Some sensors systems unique to a single study include custom smartphone networks in designated workplaces, Wi-Fi-based meshes in malls hosting guest Wi-Fi, and pressure-sensitive flooring in a smart home setting.

Almost half (14 of 28) of the studies using IR used the CASAS Project system [95], which involved a combination of IR sensors and additional switch state sensors on appliances and doors. One study [33] used data from the CASAS Project database to perform comparison analysis with their own IR-based tracking system. Within studies using IR, two studies did not specify if they used passive or active infrared [4,63].

Overall, 44 studies (55.7%) used systems such as RFID, passive IR, or state-switch sensors, which trigger “motion events” to denote the location of the study subject in time only as they pass through a fixed sensor location to activate a sensor event. Data from these devices typically have non-uniform sampling times and may have large gaps in recordings, where it is assumed that no change in location occurs. Eleven of the studies using these technologies focused on activity recognition (*n* = 13).

About half (43, 54.4%) of papers only used data from RTLS sensors to achieve their study goals. The remaining 36 studies were multi-sensor and included sensors such as door switch sensors, stove temperature sensors, bed pressure sensors, accelerometers and IMUs, microphones for audio, and/or heartrate and other biometric sensors. The use of additional sensors varied based on the RTLS sensor used. For example, door or contact switches were used exclusively in studies with passive trigger-type sensors to increase the accuracy of activity recognition and within-room location prediction for RTLS systems with lower data granularity, resolution, or accuracy. In contrast, only 1 of 11 studies using UWB sensors used additional sensors, built into a robotic station, for a safety application [89]. Three studies using biometric sensors used Bluetooth for RTLS and were able to have all of the sensors on the same IoT network [85,86,87]. The only additional sensor data used in the consumer behaviour sector studying shopper behaviour were Point-of-Sales data. There was a gap in the descriptions of sensors used, with 48 studies (60.8%) failing to provide a sensor vendor name or details about whether the sensor was developed in-house, with only three vendors occurring more than once: iBeacon in two studies, X10 in three studies, and Ubisense in nine studies.

While studies specifically focusing on localization accuracy and technological improvements to the RTLS systems were excluded in this scoping review’s screening criteria, very few papers reported system accuracy or sampling rate. Of the 11 studies reporting accuracy, eight reported UWB accuracy to a range of 10–30 cm; 1 study [5] did not provide the sensor type but provided a localization error with a mean deviation of 2.28 m. None of the 42 papers that used passive IR, RFID, or state-switch sensors, which do not have steady sampling rates, reported accuracy; however, a few specified the resolution of the location dataset by defining the spacing of sensor placement [63], stating sensor activation range [83,84], or simply describing the system as having “room-level” accuracy [61]. It is worth noting that in separate papers on consumer behaviour, e.g., [67,69,72,76,77] and operational efficiency [89], RTLS systems were attached to shopping carts and parking structure vehicles, respectively, as opposed to direct tracking of the study participants.

Several of the studies made use of shared or publicly available data (22 studies; 27.8%). Data from the CASAS datasets were collected or used in 14 studies, with six of these using the Aruba testbed studies, which contained data from a single participant. A further three studies used the same dataset gathered in a hospital setting on the location data from primary care staff participants. Two sets of studies on museum consumer behaviours used data collected from the same venue: [71,72,73,74,80,81].

## 4. Discussion

This scoping review sought to consolidate existing research on the analysis of human behaviours using indoor location tracking data and identify common applications and features of RTLS data. The most common research application of RTLS data for measuring human behaviours was in the area of monitoring or assessing health status, and in particular, cognitive impairment in older adults. We were able to categorize features derived from spatiotemporal RTLS data into four groups: proximities, trajectories, activities, and dwell time. RTLS data was most often analysed to provide measures of activity level and very few features used the proximities of two measured locations at once. Many different location-based sensor types were used to varying success and about half used additional non-RTLS sensors for more comprehensive datasets.

Overall, we found important gaps and opportunities for improvement in the analysis of indoor location data for healthcare. Many of the studies are quite preliminary in nature, only assess feasibility of using RTLS to detect patterns of activity, and without any clinical validation of their findings. A good portion of studies do not develop models for purposes beyond activity recognition and present model feasibility results for use in future studies in their respective field of study (e.g., detecting abnormal behaviours in older adults). While these systems were successfully implemented in their target end-user environments, there is a lack of evidence that the measurement of a study’s predefined set of activities are associated with any clinically meaningful patterns of human behaviour. Furthermore, in the Health Status Monitoring category, less than half of studies included evaluation against gold standard or validated clinical assessments, such as using the mini-mental state examination (MMSE) [7,23,32,54] to validate a measure of cognitive health status. Of the studies in which the success of activity recognition was a main outcome measure, none involved a clinical assessment as a comparison standard. There exists a gap in the use of RTLS models and their correlation to clinically meaningful and useable indices for behaviour or health monitoring.

For the purposes of early detection and assessing populations transitioning between different care environments, clinical validation with more longitudinal studies of independent participants with measured health declines or pre/post incidents is warranted. Few studies used multiple groups of study populations, and even fewer studies provided external validation in multiple study sites to show transferability of study paradigms. Feasibility remains an unresolved barrier. Some studies, including some earlier datasets in the CASAS databank, required participants to relocate to smart homes or apartments close to the lab adjoined to the study. Studies that investigated populations with existing health conditions required study settings where participants with acute conditions were accessible. For example, a group of studies from the University of South Florida classifying levels of cognitive impairment from trajectory patterns in location data [54,55] collected data from participants admitted to nursing homes where the sample population had a low average in MMSE scores. Models developed with subset populations (e.g., all patients with acute impairments or all healthy participants) may provide skewed predictions when applied to intermediate cases.

A notable gap in the healthcare RTLS literature is seen in the lack of analyses including features derived from the proximities between individuals. Nine (9) studies measured features of proximity, of which 2 only measured proximity to key objects. Only two studies monitoring health status used proximities between individuals, and a further three studies attempted to provide measures of “social” behaviours. The authors of [83] used the proximity of two museum visitors in a group and measured audio levels to estimate social engagement between groups using or not using mobile device guides. The two papers studying developmental behaviours used observations made by teaching staff to provide labels for RTLS data analysis [93,94]. The advanced methods in network science found in [93] were more complex in algorithms and analyses than any studies observing RTLS for health monitoring. For example, one group [47] recorded the proximity of participants to correct the accuracy of their location detection system, one [78] recorded the number and density people in one area to measure customer flow but not interaction, and one [22] measured proximity with respect to moveable objects and not other participants. While some studies [5,58,59] did not use features measuring proximity directly, dwell times in recreational or common areas were labelled separately from dwell time in private areas. The authors of Grunerbl et al. [57] recorded location data in recreational and social areas, and total time duration in these locations was investigated as a possible correlation to cognitive health status. Yang et al. [58] especially focused on measuring social isolation and privacy in LTC spaces and was the only paper to use “distance away from bedroom each day” as a feature to indicate social activity levels of study participants.

A key limitation of the reviewed studies examining social behaviour was the lack of available “ground-truth” or independent data sources for validation. While the amount of time spent in each area was measured in [57], no measure of actual engagement between multiple participants in social areas was recorded to corroborate RTLS data. The authors of Masciadri et al. [96] proposed various indices of social engagement and social isolation based on location data, and their continuing study [53] is the only study in this review with data extracted that analyse behaviours with features of proximity for assessing cognitive health status. There is a need for further investigation on whether measuring levels of social engagement solely based on social proximity and dwell time in social areas is meaningful or ill-defined. The use of multiple participants’ data at one time could be applied to high-traffic health monitoring environments such as hospitals and long-term care facilities. The use of microphones triggered when two participants are within a certain proximity to each other to measure audio level without recording conversation [83] could be considered in settings where privacy and continuous monitoring is a concern. RTLS systems could provide a solution to the lack of objective measures for social engagement in older adults in aged residential care.

Studies that are not explicitly focused on health monitoring provide some important insights that can be applied in the health monitoring space. Clinician and care staff’s perspective of the health care environment were studied in papers within the operational safety and efficiency sector [85,86,87], and insights into staff efficiency can be used to examine the quality of care received. Health monitoring algorithms that incorporate RTLS data from both healthcare staff and patients is one area of future opportunity. Behaviour prediction models developed for predicting consumer behaviours based on environmental changes provides important examples of experimental designs for assessing effectiveness of environmental interventions. This concept was introduced briefly in the long-term care home in [53]; however, there are few studies focusing on health status monitoring that compare different environments, or where the environment is manipulated in some way, and its effect on behaviours evaluated. Some studies, such as Frascella et al. [97], were excluded for lack of RTLS technology used in place of manual observation in a museum setting, but insights could still be drawn from the behaviour-analysis techniques used. These observations accentuate the necessity for exploring the wide scope of RTLS technologies used to observe human behaviours across many use cases.

One sector of RTLS applications mostly excluded during our scoping review was geographical analysis of movement outdoor locations and geographical information systems (GIS). Papers studying human behaviour from RTLS during social events [98,99] and vehicular travel [100] were identified in the Scopus search; however, as they used outdoor locations, they were excluded from final paper selection. Visualisation of spatiotemporal data in more advanced manners than simple heatmaps and binary sensor activation maps is used commonly in the GIS field [101]. Complex analyses of these visualizations present the potential to identify unique patterns of human behaviour from large datasets. Reviews of papers in the application of GIS also investigate different methods of categorizing features of RTLS data such as in [102,103], which examines parameters, properties, and factors of spatiotemporal data by complexity (primitive—e.g., distance and direction, derived—e.g., velocity, and second derivatives—e.g., spatial distribution). While some scaling may be required, techniques used in this field are relevant to describing patterns of human behaviour and could be applied to, and should be investigated in, the indoor applications identified in this scoping review.

There are a number of technologies that have location data with important meaning to health care; however, the ability to extract clinically meaningful data depends largely on how the data are collected. A number of differences in study outcomes, features, and sensor systems were found between systems using passive trigger RTLS sensor types and their higher-resolution, active-tracking counterparts. A question is raised of whether passively triggered RTLS technologies are sufficient for more complex behavioural analysis or whether the precision levels of these kinds of devices are lacking. In particular, RFID and IR technologies provide a lower level of granularity in location tracking (despite being accurate and less costly [11]), which may detract from precisely extracting within-region behaviours or continuous movement patterns such as those extracted from trajectory-type features such as path tortuosity. Conversely, UWB and other active sensors required wearable devices that need battery or recharging, whereas passive trigger-based sensors are typically wall mounted with little maintenance [11]. It is suggested that, when feasible to implement at a fair cost, using higher granularity, active sensors with a continuous data stream will provide more opportunity for complex analysis of human behaviours from RTLS data. Additionally, where lower-granularity RTLS sensors are used, it may be helpful to combine passive sensor modalities that do not interfere with daily living (e.g., door sensors) to augment the quality of the data and increase the depth of analyses that can be performed. Previous review literature reinforces the existence of a trade-off between implementation costs and data precision and richness [10,11]. The level of in-depth or basic analysis varied widely between studies and subsequently creates a variation in the applicability of study results to their target use case.

Alternatively, one method observed for increasing information gained from passive sensors was to map the spatial relation between each of the RTLS sensors in a floor plan. Four (4) of the 17 papers that used a feature defined as activity level measured by the “number of raw sensor activation triggers” developed “binary motion density maps” or “heat maps” along with their trigger-type sensors: three [7,46,47] used only levels of sensor activation in a similar manner to indicate changes in health status and provide alerts, and one [50] used DCNN with the maps but only to recognize different activities based on location as a method of remote monitoring. By using maps correlated to the floor plan of the study sites, these papers were able to re-incorporate the otherwise lost relationship between the location of each sensor with respect to the distance between them.

### Limitations and Future Work

The findings of this review may be limited by use of the Scopus database as the sole source for feasibility reasons given the broad scope of the search. Scopus was chosen due to its size, comprehensive and multi-disciplinary nature, and its inclusion of peer-reviewed conference papers. Scopus encompasses the MEDLINE and EMBASE indices and provides a citation searching feature that was useful to identify further papers. A preliminary examination of other indices did not reveal any systemic gaps in our search, but it is possible that some studies were missed.

With 79 papers, the variation between all components of the papers made statistical meta-analysis unfeasible to perform and was beyond the remit of a scoping review. Results and findings from the selected papers could not be numerically compared due to the vast differences in goals, metrics, sensor types, and methods of representation. The varying number of studies in each application sector and analysis methodology limited the interpretation of these findings. While the purpose of this work was to provide a scoping review of RTLS systems for analysing behaviour in any application, and transferable knowledge was gained from non-health-based fields, future work might focus on a narrower research question followed by meta-analysis.

## 5. Conclusions

In this scoping review, we identified 79 studies examining the use of RTLS data to describe aspects of human behaviour, with the most common application goal to monitor health status, followed by analysing consumer behaviours, increasing safety and operational efficiency, and investigating developmental child behaviours. Activity level, dwell time, trajectory, and proximity features were used to help describe these behaviours, with the level of complexity of analysis dependent on the types of sensors used. We identified a need for the collection of validated measures to serve as gold standards and a need to move beyond correlation to predictive modelling of these behaviours. While it is evident that RTLS technologies provide valuable longitudinal spatiotemporal data and can be a useful tool in analysing patterns of human behaviours, it is necessary for future studies to incorporate more complex feature analysis methods to extract the richness of location-based datasets.

## Figures and Tables

**Table 1:** Search term concepts used within the Scopus Database.

Search terms include three different concepts of location, indoor, and behaviour:
Location: real-time locating system and RTLS, geographic locations, location monitoring, geographic monitoring. indoor position, indoor positioning, sensor network, sensor data, sensor technology, motion sensor, motion density, motion mapping, motion tracking, tracking device, location management, motion density map
Indoor: indoor, school, childcare, long-term care, nursing home, residential facilities, community-dwelling, nursing facilities, hospital, shopping center, mall, site, retail store, school, classroom, warehouse, house, home, inside, inpatient, healthcare environment, daycare, living environment
Behaviours: task analysis, behavior analysis, behavior research, behavior pattern, digital phenotyping, shopper behavior, health status, smart health, agitation, wandering behaviors, ambulation, depression, life-space assessment, operations research, provider scheduling, pathways, lean management, production control, value adding time, walking path, stay time, spatiotemporal, dementia, behavior assessment, behavior monitoring, health assessment, health monitoring, health analysis, health pattern, task assessment, task pattern, task monitoring

**Table 2:** Details of the 79 studies included in scoping review analysis.

Reference	Objective	Environment	Population	Sensor	Feature Categories
Health Status Monitoring					
Judah 2017 [22]	To develop and test a reliable RTLS system that can recognize various bathroom activities and behaviours of multiple individuals	Bathroom	Not Given	Combo (Elpas)	Trajectory, Proximity
Kaye 2012 [23]	To examine the relation between measures of walking activity and function	Private Home	Adults	IR	Activity Levels
Hayes 2008 [24]	To find distinguishable differences in the motor activity of healthy and cognitively impaired elders	Private Home	Older Adults	IR	Activity Levels
Lymberopoulos 2011 [25]	To develop a model that describes and determines a person’s routine based on their spatiotemporal activity	Private Home	Older Adults	IR	Dwell, Trajectory
Petersen 2014 [26]	To describe and validate a method for detecting time spent out-of-home using a logistic regression-based classifier with inputs derived from passive sensor data.	Private Home	Older Adults	IR	Activity Levels
Fiorini 2017 [27]	To describe and define groups of behavioural patterns starting from unannotated data analysis and a “blind” approach for activity recognition	Private Home	Older Adults	IR	Activity Levels
Enshaeifar 2018 [28]	To develop an algorithm that identifies daily routines, detects unusual patterns and possible agitation events	Private Home	Older Adults	Pressure	Activity, Trajectory
Akl 2015 a [6]	To explore the feasibility of autonomously detecting mild cognitive impairment (MCI) using various features of location-tracked data	Private Home	Older Adults	IR	Activity Levels
Akl 2015 b [29]	To detect mild cognitive impairment using differences in walking speed distributions	Private Home	Older Adults	Not Given	Activity levels
Akl 2016 [30]	To automatically detect MCI in older adults using the distribution of activity in different rooms of the home	Private Home	Older Adults	IR	Activity levels
Akl 2017 [31]	To develop models of home activity that can support early detection of dementia	Private Home	Older Adults	IR	Dwell, Trajectory
Dodge 2012 [32]	To test if the assessment of walking speed and its variability can distinguish those with mild cognitive impairment (MCI) from those with intact cognition	Private Home	Older Adults	IR	Activity, Dwell
Yahaya 2019 [33]	To develop a method of finding thresholds for abnormalities in Activities of Daily Living (ADL) correlated to changes in sleeping behaviour	Private Home	Adults	IR; CASAS	Activity Levels
Tan 2018 [34]	To develop a novel DCNN classifier to recognize different activities in a smart home	Private Home	Adults	CASAS	DCNN Classifier
Gochoo 2019 [35]	To develop an unobtrusive activity recognition classifier using deep convolutional neural network (DCNN)	Private Home	Adults	CASAS	DCNN Classifier
Xu 2020 [36]	To compare different classification algorithms in their ability to recognize the at-home activity of elderly people	Private home	Older Adults	CASAS	Activity Levels
Eisa 2017 [37]	To detect unusual changes in regular mobility behaviour by monitoring daily room-to-room transitions and permanence habits	Private Home	Older Adults	CASAS	Activity, Dwell, Trajectory
Gochoo 2017 b [38]	To classify walking/travel patterns of elderly people living alone using a Deep Convolutional Neural Network classifier (DCNN)	Private Home	Older Adults	CASAS	Activity, Dwell, Trajectory
Gochoo 2017 c [39]	To develop a Deep Convolutional Neural Network (DCNN) classifier for elderly activity recognition	Private Home	Older Adults	CASAS	Activity Levels
Zhang 2017 [40]	To propose an unsupervised learning approach that can determine movement patterns and daily activities without event annotations	Private Home	Older Adults	CASAS	Trajectory
Fang 2020 [41]	To locate and predict the position of the elderly, helping to detect the abnormal behaviours or irregular life routines	Private Home	Adults	State-change Sensors	Trajectory
Fahad 2013 [42]	To monitor the change in the repeated group of activities that make up the daily routine of a person living in a smart home	Private Home	Adults	State-change Sensors	Activity, Trajectory
Su 2018 [43]	To build an activity recognition system for elder persons with dementia via the classification of hand movements and indoor position data	Smart Home	Not Given	Bluetooth	Random Forest Model
Li 2017 [44]	To test a system for screening elders who are likely to have dementia from performing eight activities from IADL	Smart Home	Older Adults	CASAS	Activity, Trajectory
Aramendi 2018 [45]	To evaluate the correlation of different behavioural features derived from daily activities to IADL-C scores and their effectiveness in detecting change in functional health decline	Smart Home	Older Adults	CASAS	Activity Levels
Rantz 2011 [46]	To investigate the use of passive monitoring of residents to detect early signs of illness, functional decline, and/or urinary tract infection	Retirement Community	Older Adults	IR	Activity Levels
Skubic 2015 [47]	To exploring behavioural features that are more or less useful in detecting early changes in health status across different chronic health conditions and home layouts	Retirement Community	Older Adults	IR	Activity, Dwell, Proximity
Galambos 2013 [7]	To investigate whether visual features from motion density maps are sensitive enough to detect changes in mental health over time	Retirement Community	Older Adults	IR	Activity, Dwell
Alberdi 2018 [48]	To evaluate use activity behaviour data to detect the multimodal symptoms that are often found to be impaired in Alzheimer’s Disease (AD) and predict related clinical scores	Retirement Community	Older Adults	CASAS	Activity Levels
Dawadi 2016 [49]	To evaluate the effectiveness of an algorithm that can model daily activity routines and detect changes in behavioural routines	Retirement Community	Older Adults	CASAS	Activity, Trajectory
Gochoo 2017 a [50]	To develop an algorithm that determines what activity is occurring at the front door and detect memory lapses (forget events from brief-return-and-exit at door)	Retirement Community	Older Adults	CASAS	Activity, Dwell, Trajectory
Tan 2017 [51]	To classify front-door events (exit, enter, visitor, other, and brief-return-and-exit) of a resident in the smart house	Retirement Community	Older Adults	CASAS	Activity, Dwell, Trajectory
Cheng 2019 [52]	To estimate dementia conditions based on graph representations of daily locomotion	Assisted Living	Older Adults	UWB	Trajectory
Bellini 2020 [53]	To assesses both the degree of relations among residents and the popularity of the facility spaces as an indicator of accessibility	Assisted Living	Older Adults	Bluetooth	Proximity
Kearns 2010 [54]	To explore whether elders with greater path tortuosity (irregular movement) was associated with greater cognitive impairment	Assisted Living	Older Adults	UWB	Trajectory
Kearns 2012 [55]	To investigate whether variability in voluntary movement paths would be greater in the week preceding a fall compared with non-fallers	Assisted Living	Older Adults	UWB	Activity, Trajectory
Bowen 2016 [9]	To examine how intraindividual changes in ambulation characteristics may be used to predict falls.	Assisted Living	Older Adults	UWB	Activity Levels
Bowen 2018 [8]	To determine the influence of cognitive impairment (CI), gait quality, and balance ability on walking distance and speed	Nursing Home	Older Adults	UWB	Activity Levels
Bowen 2019 [56]	To examine the characteristics of wandering associated with preserved versus worsened ADL function.	Nursing Home	Older Adults	UWB	Activity Levels
Grunerbl 2011 [57]	To develop and evaluate a system for coarse assessment of the health status of dementia patients in a nursing home	Nursing Home	Older Adults	UWB	Activity, Dwell
Jansen 2017 [5]	To provide descriptive analysis of life-space movement patterns in nursing home residents and to identify associated factors of different patterns	Nursing Home	Older Adults	Not Given	Activity, Dwell
Yang 2020 [58]	To classify probable social interaction patterns and identify mobility patterns and associated levels of privacy with both social and movement patterns	Nursing Home	Older Adults	Bluetooth	Activity, Dwell, Trajectory
Okada 2019 [59]	To predict scores on the dementia scale using behavioural features as observed through human–robot interactions and indoor daily activity	Nursing Home	Older Adults	Bluetooth	Dwell Time
Ramezani 2019 [60]	To examine the ability of combination of physical activity and indoor location features to discriminate subacute care patients who are re-admitted to the hospital	Inpatient Unit	Older Adults	Bluetooth	Activity, Dwell
Vuong 2014 [61]	To determine an automated system for detecting and classifying travel patterns in people with dementia using movement data	Inpatient Unit	Older Adults	RFID	Trajectory
Jeong 2017 [4]	To assess the feasibility of using an infrared-based RTLS for measuring patient ambulation in a 2-min walk test (2MWT)	Inpatient Unit	Adults	IR	Activity Levels
Kearns 2016 [62]	To determine if improvements in cognitive function during traumatic brain injury treatment can be measured using movement path tortuosity in everyday ambulation	Inpatient Unit	Adults	UWB	Trajectory
Jeong 2020 [63]	To evaluate novel ambulation metrics in predicting 30-day readmission rates, discharge location, and length of stay of postoperative cardiac surgery patients	Inpatient Unit	Cardiac Patients	IR	Activity, Dwell
Consumer Behaviour					
Dogan 2019 [64]	To show the potential of process mining techniques to understand customer needs and behavioural trends based on gender differences	Shopping Mall	Shoppers	Bluetooth	Trajectory
Liu 2020 [65]	To produce a method to infer customer profiles, mainly gender and age, using indoor location data	Shopping Mall	Shoppers	WiFi	Activity, Dwell, Trajectory
Dogan 2020 [66]	To use process mining to determine customer visit time and describe different customer flows between customers who purchase and those who do not	Supermarket	Shoppers	Bluetooth	Dwell, Trajectory
Kholod 2011 [67]	To examine grocery shoppers’ moving direction within the store and its influence on their buying behaviour	Supermarket	Shoppers	RFID	Trajectory
Popa 2013 [68]	To develop a framework for automatic assessment of customers’ behaviours to categorize them into different shoppers’ types by goal	Supermarket	Shoppers	Camera	Trajectory
Paolanti 2017 [69]	To model and predict shopper’s behaviour in retail environments to predict the shopper’s trajectory	Supermarket	Shoppers	UWB	Activity, Dwell, Trajectory
Yang 2019 [70]	To define the relationship between the layout of the shelves, and shopping behaviour and product sales	Supermarket	Students	UWB	Activity, Dwell
Takai 2010 [71]	To describe the relation between the time customers spend in a store section and the probability they will make a purchase	Supermarket	Shoppers	RFID	Dwell Time
Takai 2011 [72]	To correlate the number of purchased items by stationary time and find a two-category model that groups shopper behaviours using this correlation	Supermarket	Shoppers	RFID	Dwell Time
Takai 2012 [73]	To capture dependencies among variables that describe purchasing behaviour based on section of stores	Supermarket	Shoppers	RFID	Dwell Time
Takai 2013 [74]	To find homogeneous groups of customers based on the number of purchased items and determine whether time period that the customer shops influences this group classification	Supermarket	Shoppers	RFID	Dwell Time
Kaneko 2018 [75]	To build a purchase behaviour model of customers and predict whether the customer will make a purchase or not	Supermarket	Shoppers	RFID	Dwell Time
Nakahara 2012 [76]	To propose models that clarify the relationship between product zone visit sequences and shopping behaviour and use them to characterize high-value purchasing customers and low-value purchasing customers	Supermarket	Shoppers	RFID	Activity, Dwell, Trajectory
Zuo 2015 [77]	To improve methods of predicting whether a customer will make a purchase or not	Supermarket	Shoppers	RFID	Dwell Time
Li 2016 [78]	To study relationships between different variables derived from the amount of time spent in different areas of the store, how much was purchased from each area, and the area type	Supermarket	Shoppers	RFID	Activity, Dwell
Gu 2019 [79]	To measure differences in product search behaviour and search benefits depending on the customer and their varying levels of self-control	Supermarket	Shoppers	RFID	Dwell Time
Yoshimura 2014 [80]	To identify aspects of visitor behaviour that could explain museum overcrowding	Museum	Museum Visitors	Bluetooth	Activity, Dwell, Trajectory
Yoshimura 2019 [81]	To compare museum visitor movements when more or fewer choices are offered	Museum	Museum Visitors	Bluetooth	Dwell, Trajectory
Kanda 2007 [82]	To estimate visitor trajectories to analyse space, visiting patterns, and relationships	Museum	Museum Visitors	RFID	Activity, Dwell, Trajectory, Proximity
Lanir 2013 [83]	To compare the movement of museum visitors who used a mobile multimedia location-aware guide to those who did not	Museum	Museum Visitors	RFID	Activity, Dwell, Proximity
Martella 2017 [84]	To understand the behaviour of museum visitors and the attraction power of different displays	Museum	Museum Visitors	RFID	Dwell, Trajectory, Proximity
Safety and Operational Efficiency					
Booth 2019 [85]	To develop a technique for clustering room purpose based on patterns in human movement data and to predict mental wellness levels of hospital staff	Hospital	Primary Care Staff	Bluetooth	Dwell, Trajectory
Feng 2020 a [86]	To detect and discover location-driven routines and physiological data to understand the movement intensity of nurses at different times in a work shift	Hospital	Nurses	Bluetooth	Dwell, Trajectory
Feng 2020 b [87]	To develop a method to quantify the relations between physiological signals and indoor locations at a real-world workplace. The method is validated on individuals’ workplace performance in a large hospital setting.	Hospital	Nurses	Bluetooth	Dwell Time
Lopez-de-Teruel 2017 [88]	To provide a method to differentiate location data of employees from non-employees and generate clusters related to the different working teams	Office	Workers	Custom wireless network + cell phones	Activity Levels
Cheng 2013 [89]	To design and validate a new method to analyse the spatio-temporal conflicts between workers and automatically defined hazard, and define an indicator that can measure the safety performance of workers	Construction	Workers	UWB	Activity, Dwell
Arslan 2018 [90]	To develop a model that uses worker mobility patterns to identify unsafe worker behaviours	Construction	Workers	Bluetooth	Activity, Trajectory
Arslan 2019 [91]	To test if semantic trajectories can visualize site-zone density to avoid congestion and provide proximity analysis to prevent collisions, accidents, and unauthorized access	Construction	Workers	Bluetooth	Activity, Dwell, Trajectory
Hwang 2019 [92]	To monitor pedestrian flow in a subway station and use sensor-based insights to improve pedestrian flow	Subway Station	Subway Commuters	Bluetooth/ Wi-Fi	Activity Levels
Developmental Behaviour					
Jorge 2019 [93]	To develop and validate an algorithm that detects unusual social behaviour and finds significant subgroups within the population	School Playground	Children	Set 1—IMU GNSS, Set 2—UWB	Proximity
Messinger 2019 [94]	To investigate differences in social interaction and movement within a classroom based on gender and describe the classroom social network	Classroom	Children	UWB	Activity, Trajectory, Proximity

## Data Availability

Additional detailed results can be found in the Supplementary file of this article.

## Supplementary Materials

The following are available online at https://www.mdpi.com/article/10.3390/s22031220/s1, Table S1: RTLS Feature Categories and References.
